# An Unusual Conversion of Paramagnetic [3-Cl-3,3,8-{Ph_2_P(CH_2_)_n_PPh-µ-(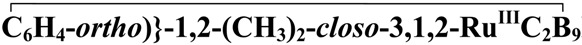
H_8_] (*n* = 3 and 4) to Form the First 18-Electron P-Phenylene *ortho*-Cycloboronated *closo-*Ruthenacarboranes with a Dioxygen Ligand

**DOI:** 10.3390/molecules19067094

**Published:** 2014-05-30

**Authors:** Alexander Y. Kostukovich, Dmitrii I. D’yachihin, Fedor M. Dolgushin, Alexander F. Smol’yakov, Ivan A. Godovikov, Igor T. Chizhevsky

**Affiliations:** A.N. Nesmeyanov Institute of Organoelement Compounds of the RAS, 28 Vavilov Street, 119991 Moscow, Russia; E-Mails: dftblyp@gmail.com (A.Y.K.); dyachihin@list.ru (D.I.D.); fedya@xrlab.ineos.ac.ru (F.M.D.); rengenhik@gmail.com (A.F.S.); pr0vider@ineos.ac.ru (I.A.G.)

**Keywords:** ruthenacarboranes, oxygen-type ligands, NMR-spectra, X-ray diffraction

## Abstract

Treatment of [3-Cl-3,3,8-[Ph_2_P(CH_2_)_n_PPh-µ-(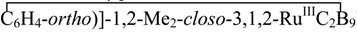
H_8_] (**1**, *n* = 3; **2**, *n* = 4) with an excess of KOH in a 1:1 benzene/methanol mixture at room temperature in air affords new dioxygen *closo-*ruthenacarborane complexes [3-(η^2^-O_2_)-3,3,8-[Ph_2_P(CH_2_)_n_PPh-µ-(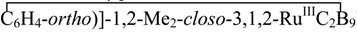
H_8_] (**3**, *n* = 3) and (**4**, *n* = 4) in 42.5% and 45.8% yield respectively. The structures of dioxygen complexes **3** and **4** were established by single-crystal X-ray diffraction. The IR and multinuclear NMR data [^1^H, ^13^C{^1^H}, ^31^P{^1^H} and ^11^B{^1^H}] along with 2D HSQC correlation spectra for the new dioxygen *closo-*ruthenacarboranes are discussed.

## 1. Introduction

Boron-containing transition metal clusters exhibiting *ortho*-cycloboronation of the phosphine or diphosphine ligands on a metal atom are long known in metallaborane [1,2,3] and monocarbon metallacarborane [4,5] chemistry. However, first examples of related 12-vertex dicarbon metallacarborane complexes were reported only recently [6,7,8,9,10,11], and all known complexes of these series are Ru^III^ species having a 17-electron shell. In this regard it is of interest that these paramagnetic *ortho*-cycloboronated metallacarborane systems demonstrate the exceptionally high efficiency as catalyst precursors for the atom transfer radical polymerization (ATRP) of methyl methacrylate, as well as of other vinyl monomers [7,8,9,10,11,12,13,14]. On the other hand, such electron-deficient complexes hold considerable promise in a variety of ways for the development of ruthenacarborane chemistry. We now report an initial study on the reactivity of paramagnetic *ortho*-phenylenecycloboronated *closo-*ruthenacarboranes resulting in preparation of a short series of new dioxygen ruthenium-carborane complexes.

## 2. Results and Discussion

Polyhedral contraction reactions of transition metals metallacarboranes occurring under base-mediated conditions to produce smaller metallacarborane species have not been extensively reported [15,16,17,18]. Hence, we proposed to extend studies in this area starting from the previously untested *ortho-*cycloboronated P-phenylene paramagnetic species [3-Cl-3,3,8-[Ph_2_P(CH_2_)_n_PPh-µ-(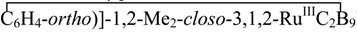
H_8_] (1,2-dimethyl-3-chloro-3,8-µ-{*ortho*-[phenyl-(3'-diphenylphosphinopropyl-κ*P*(Ru))phosphino]phenyl-*P*(Ru),*C*^1^(B^8^)}-1,2-dicarba-3-ruthena-*closo*-dodecaborane (**1**) *n* = 3 [7] and 1,2-dimethyl-3-chloro-3,8-μ-{*ortho*-[phenyl-(4'-diphenylphosphinobutyl-κ*P*(Ru))phosphino]phenyl-*P*(Ru),*C*^1^(B^8^)}-1,2-dicarba-3-ruthena-*closo-*dodecaborane (**2**); *n* = 4 [9]) with the hope of preparing either *ortho-*cycloboronated 11-vertex {RuC_2_B_8_} complexes or smaller clusters of these series. Accordingly, we examined the reaction of **1** and **2** with a fourfold molar excess of KOH at room temperature in a 1:1 benzene/methanol mixture in air.

**Scheme 1:**
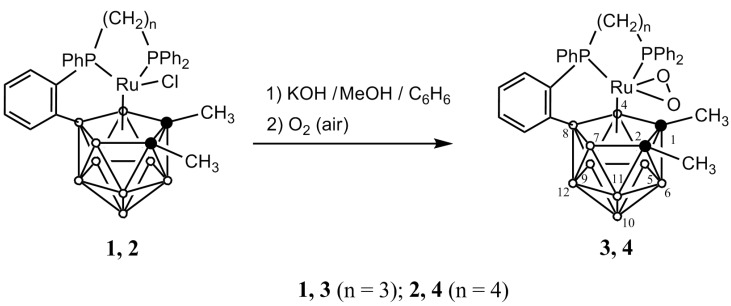
Schematic representation of the reactions of **1** and **2** with an oxygen.

Only ruthenacarborane products were isolated from these reactions, each in *ca.* 45% yield, after column chromatography on silica gel. These species proved, however, to exhibit different structures from those anticipated. It was evident from crystallographic data that the isolated species are actually novel 18-electron diamagnetic *closo*-ruthenacarboranes with the dioxygen ligand at the metal vertex, *viz*., [3-(η^2^-O_2_)-3,3,8-[Ph_2_P(CH_2_)_n_PPh-µ-(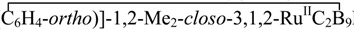
H_8_] (**3**, *n* = 3; **4**, *n* = 4), respectively (Scheme 1).

**Figure 1:**
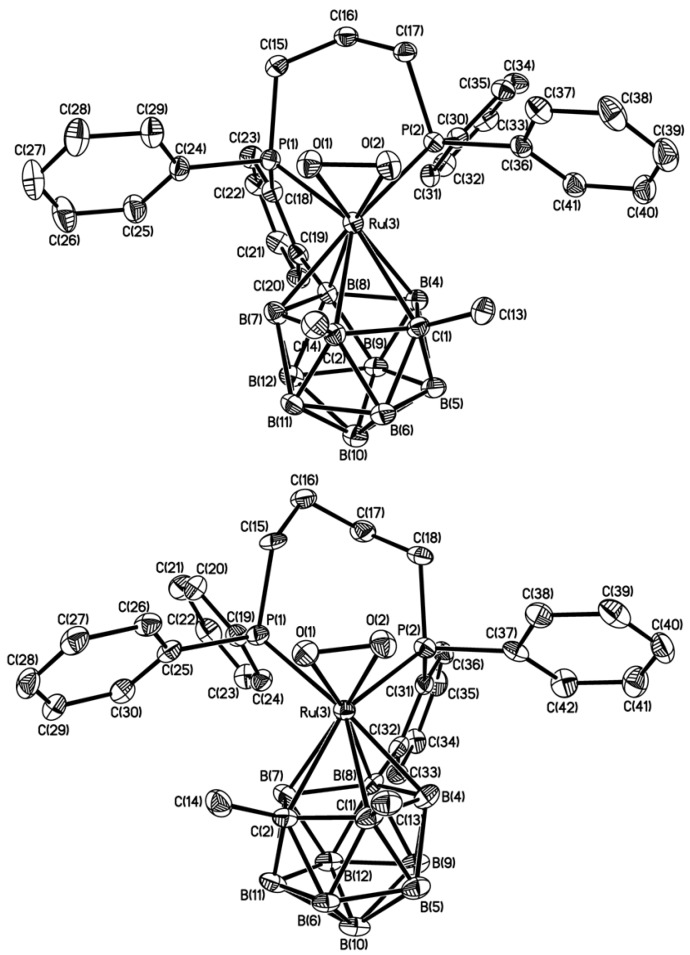
ORTEP representation of the molecular structures of complexes **3** (top) and **4** (bottom) with thermal ellipsoids at the 50% probability level. Hydrogen atoms are omitted for clarity.

Single-crystal X-ray diffraction studies were carried out on both species **3** and **4**. Their selected bond distances and angles are summarized in Table 1. Figure 1, on the top, shows the molecular structure of complex **3**. In the crystal of complex **4**, there are two independent molecules, which show only small differences in their geometry (see Table 1), and the molecular structure of only one of them is therefore shown in Figure 1, on the bottom.

**Table 1:** Selected bond lengths (Å) and angles (deg.) in **3** and **4** (two independent molecules A and B).

Compound	3	4	
		A	B
Ru(3)-O(1)	2.037(2)	2.036(3)	2.015(3)
Ru(3)-O(2)	2.040(2)	2.041(3)	2.043(3)
Ru(3)-P(1)	2.3412(7)	2.3580(13)	2.3693(13)
Ru(3)-P(2)	2.2928(7)	2.3201(13)	2.3280(13)
Ru(3)-C(1)	2.320(3)	2.316(5)	2.318(5)
Ru(3)-C(2)	2.336(3)	2.356(5)	2.338(4)
Ru(3)-B(4)	2.251(3)	2.255(5)	2.282(5)
Ru(3)-B(7)	2.251(3)	2.258(5)	2.290(6)
Ru(3)-B(8)	2.253(3)	2.259(5)	2.274(5)
O(1)-O(2)	1.403(3)	1.399(4)	1.404(4)
C(1)-C(2)	1.621(4)	1.633(7)	1.640(6)
O(1)-Ru(3)-O(2)	40.26(8)	40.14(12)	40.49(12)
O(1)-Ru(3)-P(2)	109.21(6)	112.24(10)	115.91(10)
O(2)-Ru(3)-P(2)	79.80(6)	81.07(10)	83.70(9)
O(1)-Ru(3)-P(1)	75.32(6)	74.19(9)	74.10(9)
O(2)-Ru(3)-P(1)	100.35(6)	98.09(10)	95.59(10)
P(2)-Ru(3)-P(1)	86.76(3)	89.17(4)	86.97(5)
O(2)-O(1)-Ru(3)	69.97(11)	69.76(18)	68.68(18)
O(1)-O(2)-Ru(3)	69.77(11)	70.10(18)	70.82(18)

According to the crystallographic data, the ruthenium atom in both **3** and **4** is bound in a η^5^-fashion to the C_2_B_3_ open face of C,C'-dimethylated *nido*-{C_2_B_9_}-carborane cage ligands, which have *ortho-*phenylenecycloboronated linkages connecting one of the P-phenyl rings of the ruthenium-bound dppp or dppb ligand, respectively, to the cluster B(8) atom. As expected, the presence of *ortho*-cycloboronated bridging fragment in both **3** and **4** accounts for the observed differences in the Ru-P(1) and Ru-P(2) bond lengths (2.3412(7) and 2.2928(7) Å in **3**; av. 2.3637(13) and 2.3241(13) Å in **4**), respectively. In addition, the metal atom in both structures **3** and **4** is coordinated by the dioxygen ligand in almost symmetrical η^2^-fashion with Ru-O distances range 2.015(3)-2.043(3) Å (av. 2.035 Å). The O-O distance in **3** (1.403(3) Å) and in **4** (1.399(4), 1.404(4) Å) is comparable to those found in the known oxygen Cp* ruthenium complexes such as [Cp*Ru(O_2_)(dppe)]PF_6_ (1.398(5)) Å [19]) or [Cp*Ru(O_2_)(dppm)]BPh_4_ (1.37(1)) Å [20], however, is somewhat shorter as compared with that in the only structurally studied oxygen iridium-carborane species with the *exo*-polyhedrally bound metal atom, [(Ph_3_P)_2_IrO_2_(7,8-(MeS)_2_-7,8-C_2_B_9_H_10_)]•Me_2_CO (1.471(8) Å) [21]. The carborane C-C bond distances in **3** (1.621(4) Å) and **4** (1.633(7) and 1.640(6) Å), as well as the other cluster C-B (1.69–1.75 Å) and B-B (1.73–1.84 Å) connectivities, are typical of 12-vertex *closo-*metallacarboranes [22].

**Figure 2:**
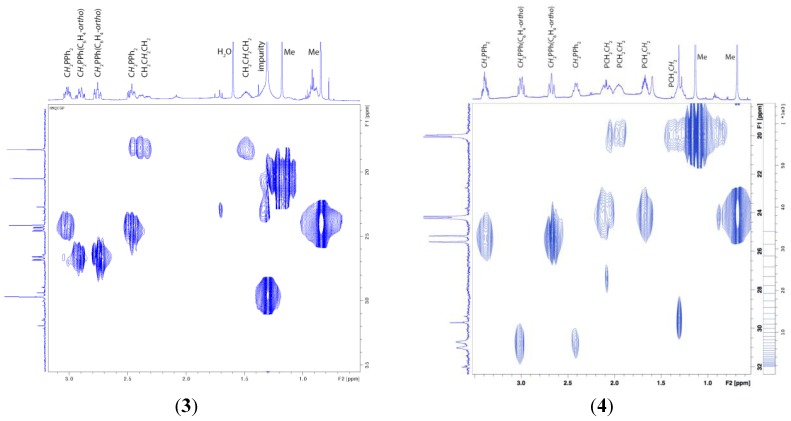
[^1^H-^13^C] HSQC spectrum for **3** (on the left) and **4** (on the right) in CD_2_Cl_2_ solution at 25 °C showing the assignment of the proton resonances.

Analytical, IR and NMR spectroscopic data are entirely consistent with the formulation of complexes **3** and **4** made based on the above X-ray diffraction experiments. The Nujol mull infrared spectra of **3** and **4** show a broad band at *ca.* 940 cm^−1^. Since no such bands were observed in the IR spectra of starting complexes **1** and **2**, these bands in IR spectra of **3** and **4** were assigned to ν_O-O_. The detailed examination of the ^1^H, ^13^C{^1^H}, and ^31^P{^1^H} NMR spectra of complexes **3** and **4** provided additional useful information on their structures. The ^31^P{^1^H} NMR spectra of complexes **3** and **4** each contains two resonances: two sharp doublets (δ_P(1)_ = 18.6 ppm, δ_P(2)_ = 52.9; *J*_P(1)-P(2)_ = 75.3 Hz) for **3**; one broadened and one sharp doublet (δ_P(1)_ = 23.0 ppm, δ_P(2)_ = 64.8; *J*_P(1)-P(2)_ = 65.0 Hz) for **4**. The low-field phosphorus resonances at δ 52.9 and 64.8 ppm were tentatively assigned to the “PPh-µ-(C_6_H_4_-*ortho*)” moiety of **3** and **4**, which is connected to boron atom of the cage ligand. The ^1^H-NMR spectra of **3** and **4**, along with the resonances derived from the phenyl protons of the dppp and dppb ligands at δ between *ca.* 7.8 and 6.8 ppm, as well as the resonances of the cage CH_3_ protons (δ, 1.14, 0.81 for **3** and δ, 1.09, 0.64 ppm for **4**), are indicative of a set of non-equivalent protons of three and four methylene groups attributable to the chelating diphosphine ligands. The latter sets proved to be the only structurally significant and are particularly diagnostic of the presence the *ortho*-phenylenecycloboronated moiety in **3** and **4**. Therefore, we strictly assigned all these resonances by the use of ^1^H{^31^P}, ^13^C{^1^H} NMR, as well as 2D [13C-1H]-HSQC correlation spectra. Thus, the fully ^31^P-decoupled ^1^H{^31^P} NMR spectra of **3** and **4** each revealed two pairs of apparently simplified 1H multiplet resonances originating from diphosphine CH_2_ groups substituted by the PPh_2_ functions. In agreement with this, in the 2D [13C-1H]-HSQC spectrum of **3** (Figure 2, on the left) each pair of these 1H multiplets are connected by cross-peaks to one of the two carbon resonances that appear as doublet of doublets at δ 24.4 and 26.7 ppm with *ca.* *J*_C,P_ = 35.3 and *J*_C,P_ = 7.5 Hz. Similar cross-peaks between each pairs of 1H multiplets, which are susceptible to ^31^P-decoupling, a broadened doublet at δ 30.8 ppm and a sharp doublet at 25.3 ppm both having *ca.* same *J*_C,P_ = 32.0 Hz can be observed in the 2D [^13^C-^1^H]-HSQC spectrum of **4** (Figure 2, on the right). The differences in the multiplicity observed for the carbon resonances derived from “Ph_2_P_(1)_*C*H_2_(CH_2_)_n_*C*H_2_P_(2)_Ph-µ-(C_6_H_4_-*ortho*)” units in the ^13^C{^1^H} NMR spectra of **3** and **4** can be explained by a shorter carbon chain in the case of diphosphine ligand of **3**, which results in additional splitting of resonances derived from *C*H_2_ groups on the far-located ^31^P nucleus.

## 3. Experimental

### 3.1. General Information

Starting complexes **1** [7] and **2** [9] were prepared according to the published procedures. Chromatography columns (*ca.* 15 cm in length and 3.5 cm in diameter) packed with silica gel (Acros, Geel, Belgium, 0.035–0.070 mm, 60 A) were used for purification of the prodacts **3** and **4**. The ^1^H, ^31^P{^1^H}, ^13^C{^1^H}, ^11^B/^11^B{^1^H} and correlation NMR spectra were obtained with a Bruker AMX-400 spectrometer (^1^H, 400.13 MHz; ^31^P, 161.98 MHz; ^13^C, 100.61 MHz; ^11^B, 128.33 MHz) using TMS as an internal reference and 85% H_3_PO_4_ and BF_3_•Et_2_O as the external references, respectively. IR spectra were obtained from nujol mull on a Nicolet Magna-750 spectrometer. Microanalyses were performed by Analytical Laboratory of the Institute of Organoelement Compounds of the RAS.

### 3.2. Preparation of [3-(η^2^-O_2_)-3,3,8-[Ph_2_P(CH_2_)_3_PPh-µ-(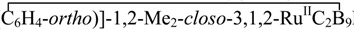
H_8_] (**3**)

A mixture of **1** (113 mg, 0.16 mmol) and an excess of crushed KOH (45 mg, 0.804 mmol) in a 1:1 benzene-methanol mixture (20 mL) was stirred at ambient temperature (~10 min) until all starting material was dissolved and the color of the solution changes from deep red to pale yellow. The flask was opened on air, and the reaction mixture was allowed to stir additionally for 1 h. Solvent was evaporated under reduced pressure and the residue was dissolved in benzene (8–10 mL), filtered and treated by column chromatography on silica gel. The brownish band was eluted from the column using benzene, the solvent was removed, and the resultant solid was re-crystallized from a *n*-hexane-benzene mixture, affording 50.8 mg (45.2% yield) of pure complex **3** as brown crystals. Anal. Calcd for C_31_H_39_B_9_O_2_P_2_Ru: C, 52.89%, H, 5.58%, B, 13.82%. Found: C, 52.82%, H, 5.56%, B, 13.70%. IR (Nujol mull, cm^−1^): ν_O-O_ 941 (w). ^1^H-NMR (CD_2_Cl_2_, 20 °C) δ 7.68 to 6.92 [set of seven m. 19H, *Ph*_2_P_(1)_(CH_2_)_3_P_(2)_*Ph*-µ-(C_6_*H*_4_-*ortho*)], 3.01, 2.46 [each m, 1H, Ph_2_P_(1)_C*H*_2_CH_2_CH_2_P_(2)_Ph-µ-(C_6_H_4_-*ortho*)], 2.90, 2.76 (each m, 1H, Ph_2_P_(1)_CH_2_CH_2_C*H*_2_P_(2)_Ph-µ-(C_6_H_4_-*ortho*)), 2.38, 1.49 [td and m, 1H, Ph_2_P_(1)_CH_2_C*H*_2_CH_2_P_(2)_Ph-µ-(C_6_H_4_-*ortho*)], 1.60 (s, H_2_O in the solvent), 1.14, 0.81 (each s, 3H, CH_3_-carb). ^31^P{^1^H}-NMR (CD_2_Cl_2_, 20 °C,) δ 52.9 (d, 1P, *J*_P,P_ = 75.3 Hz), 18.5 (d, 1P, *J*_P,P_ = 75.3 Hz). ^13^C{^1^H}-NMR (CD_2_Cl_2_, 20 °C) δ 139.1, 138.7, 135.5, 128.7 (each d, *J*_C,P_ = 58.4, 58.7, 54.4, 48.6 Hz, C_ipso_), 134.8 (br. s), 134.1 (d, *J*_C,P_ = 6.7 Hz), 134.0 (d, *J*_C,P_ = 20.0 Hz), 132.2, 130.7, 130.3, 130.2 (each d, *J*_C,P_ = 2.0, 2.7, 2.2, 2.7 Hz, C_para_), 131.2 (d, *J*_C,P_ = 7.6 Hz), 128.3 (d, *J*_C,P_ = 10.8 Hz), 127.8 (d, *J*_C,P_ = 9.9 Hz), 127.6 (d, *J*_C,P_ = 9.4 Hz), 126.7 (d, *J*_C,P_ = 8.6 Hz), 75.6 (br. d-like, C_carb_), 75.2 (br. d-like, C_carb_), 29.7 (s, Me), 26.7 (dd, *J*_C,P_ = 35.0, 7.0 Hz, *C*H_2_P_(1)_Ph_2_), 24.4 [dd, *J*_C,P_ = 35.7, 8.1 Hz, *C*H_2_P_(2)_Ph-µ(C_6_H_4_-*ortho*)], 24.1 (s, Me), 18.3 (s, PCH_2_*C*H_2_CH_2_P). ^11^B{^1^H} NMR (CD_2_Cl_2_, 20 °C): 17.2 (s, 1B), −1.8 (3B), −3.0 (1B), −4.0 (1B), −7.9 (3B).

### 3.3. Preparation of [3-(η^2^-O_2_)-3,3,8-[Ph_2_P(CH_2_)_4_PPh-µ-(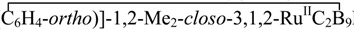
H_8_] (**4**)

The procedure was carried out as for **3**: starting complex **1** (45 mg, 0.062 mmol), KOH (17 mg, 0.304 mmol), 1:1 benzene-methanol mixture (10 mL), 10 min + 1 h under air. After isolation and purification via column chromatography on silica gel, the product was then re-crystallized from *n*-hexane–benzene, affording 20.5 mg (45.8% yield) of pure complex **4** as brownish-yellow crystals. IR (Nujol mull, cm^−1^): ν_O-O_ 938 (w). Anal. Calcd for C_32_H_41_B_9_O_2_P_2_Ru•C_6_H_6_: C, 57.33%, H, 5.95%. Found: C, 57.62%, H, 6.13%. ^1^H-NMR (CD_2_Cl_2_, 20 °C, *J*, Hz):, 7.8–6.9 [set of eight overlapped m. 19H, *Ph*_2_P_(1)_(CH_2_)_4_P_(2)_*Ph*-µ-(C_6_*H*_4_-*ortho*)], 3.35, 2.37 (m and q-like, 1H, Ph_2_P_(1)_C*H*_2_CH_2_CH_2_CH_2_P_(2)_Ph-µ-(C_6_H_4_-*ortho*)), 2.97, 2.63 (q-like and t-like, 1H, Ph_2_P_(1)_CH_2_CH_2_CH_2_C*H*_2_P_(2)_Ph-µ-(C_6_H_4_-*ortho*)), 2.05, 1.92, 1.63, 1.27 (each m, 1H, Ph_2_P_(1)_CH_2_C*H*_2_C*H*_2_CH_2_P_(2)_Ph-µ-(C_6_H_4_-*ortho*)), 1.09, 0.64 (each s, 3H, Me). ^31^P{^1^H}-NMR (CD_2_Cl_2_, 20 °C, *J*, Hz): 64.7 (d, br, 1P, *J*_P,P_ = 65.0 Hz), 22.9 (d, 1P, *J*_P,P_ = 65.0 Hz). ^13^C{^1^H}-NMR (CD_2_Cl_2_) δ 140.2 (d, *J* = 54.0 Hz, C_ipso_), 139.5, 138.9 (overlapping d, *J*_C,P_ = 51.3, 58.0 Hz, C_ipso_), 134.3 (d, *J*_C,P_ = 5.1 Hz), 133.5 (d, *J*_C,P_ = 20.4 Hz), 131.8, 130.7, 130.2, 130.0 (each d, *J*_C,P_ = 2.1, 2.1, 2.6, 2.6 Hz, C_para_), 130.9 (d, *J*_C,P_ = 7.4 Hz), 129.6 (d, *J*_C,P_ = 41.9 Hz, C_ipso_), 129.0 (d, *J*_C,P_ = 4.1 Hz), 128.3 (d, *J*_C,P_ = 9.5 Hz), 127.7 (d, *J*_C,P_ = 9.5 Hz), 126.7 (d, *J*_C,P_ = 8.1 Hz), 76.4–75.8 (m, C_carb_), 30.9 (br. d, *J*_C,P_ = 32.9 Hz, *C*H_2_P_(2)_Ph-µ-(C_6_H_4_-*ortho*)), 25.4 (d, *J*_C,P_ = 31.2 Hz, *C*H_2_P_(1)_Ph_2_), 24.3, 20.0 (each s, PCH_2_*C*H_2_*C*H_2_CH_2_P), 24.2, 20.1 (each s, Me). ^11^B{^1^H} NMR (CD_2_Cl_2_, 20 °C): 17.5 (s, 1B, B(8)), −1.0 (2B), −2.1 (1B), −3.9 (1B), −6.9 (2B), −7.8 (1B), −10.0 (1B).

### 3.4. X-ray Structure Determination for Complexes **3** and **4**

Single-crystal X-ray diffraction study was carried out with a Bruker SMART 1000 [23] for **3** and Bruker SMART APEX II [24] for **4** diffractometers (graphite monochromated Mo-K_α_ radiation, *λ* = 0.71073 Å, *ω*-scan technique). The SHELXTL software [25] was used for space group and structure determination, refinements, graphics, and structure reporting. The structures were solved by direct methods and refined by the full-matrix least-squares technique against *F*^2^ with the anisotropic thermal parameters for all non-hydrogen atoms. The hydrogen atoms of carborane moieties were located from difference Fourier maps, the rest hydrogen atoms were placed in the geometrically calculated positions. All hydrogen atoms were included in the structure factors calculations in the riding motion approximation. In the crystal structure of **4**, the central atom C (16B) of dppb ligand in the one of two independent molecules is disordered over two positions with 0.8/0.2 occupancies. In both structures **3** and **4**, the CH_2_Cl_2_ solvent molecules are presented. The principal experimental and crystallographic parameters are listed in Table 2. CCDC 992977 (for **3**) and 992978 (for **4**) contain the supplementary crystallographic data for this paper. These data can be obtained free of charge via http://www.ccdc.cam.ac.uk/conts/retrieving.html (or from the CCDC, 12 Union Road, Cambridge CB2 1EZ, UK; Fax: +44 1223 336033; E-mail: deposit@ccdc.cam.ac.uk).

**Table 2:** Crystal data, data collection and structure refinement parameters for **3** and **4**.

Compound	3	4
Molecular formula	C_31_H_39_B_9_O_2_P_2_Ru•2(CH_2_Cl_2_)	C_32_H_41_B_9_O_2_P_2_Ru•0.25(CH_2_Cl_2_)
Formula weight	873.77	739.18
Dimension, mm^3^	0.20 × 0.15 × 0.10	0.28 × 0.14 × 0.07
Crystal system	triclinic	triclinic
Temperature, K	120	100
Space group	*P*?1	*P*?1
*a*, Å	9.791(2)	10.361(2)
*b*, Å	10.063(4)	16.496(4)
*c*, Å	20.831(1)	21.721(5)
*α*, deg.	82.764(1)	76.958(4)
*β*, deg.	84.678(1)	81.790(4)
*γ*, deg.	62.696(1)	79.194(4)
*V*, Å^3^	1894.6(1)	3533(1)
*Z*	2	4
*ρ*_calc_, g·cm^−3^	1.532	1.390
2 *θ*_max_, deg.	60	54
Linear absorption ( *µ*), cm^−1^	8.13	7.95
No. unique refl. ( *R*_int_)	10975 (0.0306)	15322 (0.1018)
No. observed refl. ( *I* > 2*σ(I)*)	8254	8960
No. parameters	470	877
*R*_1_ (on *F* for observed refl.) ^a^	0.0456	0.0534
*wR*_2_ (on *F*^2^ for all refl.) ^b^	0.1017	0.1197
*GOOF*	0.980	0.907

Note: ^a^ *R*_1_ = Σ||*F_o_* − |*F_c_*||/Σ|*F_o_*|; ^b^ *wR_2_* = {Σ[*w*(*F_o_*^2^ − *F_c_*^2^)^2^]/Σ*w*(*F_o_*^2^)^2^}^1/2^.

## 4. Conclusions

The complexes described in this paper are novel oxygen-containing *ortho-*phenylenecycloboronated *closo*-ruthenacarboranes of the general formula [3-(η^2^-O_2_)-3,3,8-[Ph_2_P(CH_2_)_n_PPh-µ-(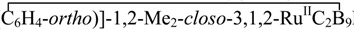
H_8_] (**3**, *n* = 3) and (**4**, *n* = 4). They were prepared by a facile and potentially useful method starting from paramagnetic species [3-Cl-3,3,8-{Ph_2_P(CH_2_)_n_PPh-µ-(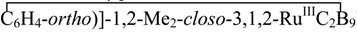
H_8_] (*n* = 3 and 4), which formally undergo a mild base-mediated chlorine-oxygen displacement reaction. Based on these and the literature data, it is envisaged that suitably constituted paramagnetic ruthenacarborane complexes as the potential precursors might open up extensive applications in cluster ruthenacarborane chemistry as well as in the homogeneous metallacarborane catalysis.
